# Differential Impacts of Alternative Splicing Networks on Apoptosis

**DOI:** 10.3390/ijms17122097

**Published:** 2016-12-14

**Authors:** Jung-Chun Lin, Mei-Fen Tsao, Ying-Ju Lin

**Affiliations:** 1 School of Medical Laboratory Science and Biotechnology, College of Medical Science and Technology, Taipei Medical University, Taipei 11031, Taiwan; 2 Department of Laboratory Medicine, Taipei Medical University Hospital, Taipei 11031, Taiwan; b8204021@tmu.edu.tw; 3 School of Chinese Medicine, China Medical University, Taichung 404, Taiwan; yjlin.kath@gmail.com

**Keywords:** alternative splicing, apoptosis, organogenesis, carcinogenesis

## Abstract

Apoptosis functions as a common mechanism to eliminate unnecessary or damaged cells during cell renewal and tissue development in multicellular organisms. More than 200 proteins constitute complex networks involved in apoptotic regulation. Imbalanced expressions of apoptosis-related factors frequently lead to malignant diseases. The biological functions of several apoptotic factors are manipulated through alternative splicing mechanisms which expand gene diversity by generating discrete variants from one messenger RNA precursor. It is widely observed that alternatively-spliced variants encoded from apoptosis-related genes exhibit differential effects on apoptotic regulation. Alternative splicing events are meticulously regulated by the interplay between *trans*-splicing factors and *cis*-responsive elements surrounding the regulated exons. The major focus of this review is to highlight recent studies that illustrate the influences of alternative splicing networks on apoptotic regulation which participates in diverse cellular processes and diseases.

## 1. Introduction

Apoptosis is defined as a death modality of damaged or unnecessary cells [1,2], which is executed by the caspase pathway [3]. The presence of proteolytic caspases triggers nuclear fragmentation, chromatin condensation, and cell rounding, and apoptotic cells are taken apart in membrane-bound vesicles [4]. Apoptotic bodies are rapidly phagocytosed by resident macrophages or neutrophils [5]. Impaired clearance of apoptotic cells leads to the exposure of intracellular organelles which frequently sensitize the innate immune system [6]. In eukaryotes, apoptosis participates in diverse processes, including immune responses [7], embryonic development [8], and maintenance of tissue homeostasis [9]. Depending on the environmental stress or cell types, apoptosis is differentially executed through extrinsic and intrinsic pathways [10,11]. The intrinsic pathway is activated in the presence of numerous intracellular stimuli, including DNA damage, endoplasmic reticular stress, oxidative stress, and breakage of mitochondrial membranes [12,13]. Activation of multiple death receptors or the withdrawal of cytokines induces extrinsic pathway-mediated apoptosis [14,15]. Both the intrinsic and extrinsic pathways lead to the release of cytochrome C and other apoptosis-inducing factors, which subsequently activate downstream caspases [16].

Alternative splicing constitutes a posttranscriptional mechanism to expand the proteomic diversity of a single gene in eukaryotes [17]. Accurate alternative splicing profiles and regulation determine cellular fates and functions [18]. It was documented that over 90% of human genes produce more than one transcript by undergoing alternative splicing mechanisms [19]. Alternative splicing profiles are meticulously regulated by the interplay among spliceosomes, splice sites, *cis*-regulatory elements, and corresponding splicing regulators, the expression profiles of which occur in a spatial-temporal manner [20]. The development of high-throughput approaches, including proteome and transcriptome analyses, has been very helpful in understanding alternative splicing mechanisms involved in cell homeostasis and pathological causes [21].

## 2. Overview of Apoptosis

**Figure 1:**
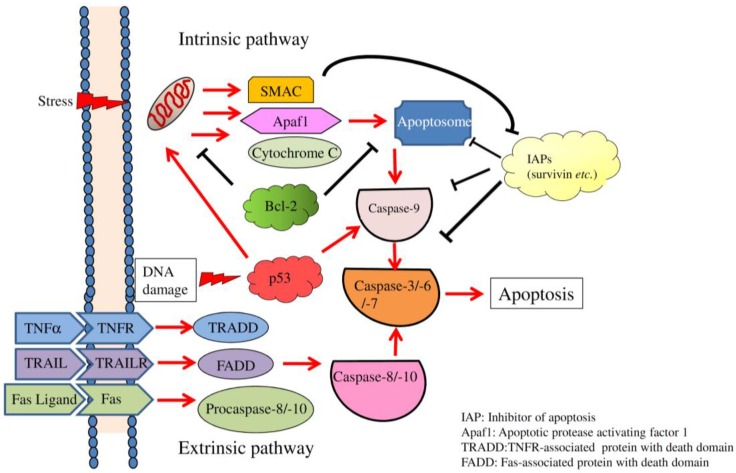
Intrinsic and extrinsic apoptosis pathways in mammalian cells. Environmental stimuli induce DNA damage or other cell stress which induces the release of second mitochondria-derived activator of caspase (SMAC), Apaf1, and cytochrome C from damaged mitochondria to form apoptosome. The presence of SMAC counteracts the repressive effect of inhibitor of apoptosis proteins, such as survivin on activate caspase-3 which acts the executer of intrinsic pathway. The extrinsic pathway is triggered by the binding of pro-apoptotic receptors and corresponding ligands that leads to the formation death-inducing signaling complex and subsequent activation of the downstream procaspases-8 and -10. TNF, tumor necrosis factor; TNFR, TNF receptor; TRAIL, TNF-related apoptosis-inducing ligand; TRAILR, TRAIL-receptor.

Apoptosis, necroptosis, and autophagy are classified as programmed cell death, an integral process to maintain a homeostatic circumstance in organisms [22]. The extrinsic and intrinsic pathways are two well-studied mechanisms that contribute to the execution of apoptosis (Figure 1; [10,11,12,13,14,15]). The binding between death ligands, including Fas ligand (Fas L), tumor necrosis factor (TNF)-related apoptosis-inducing ligand (TRAIL), and TNF-α, and corresponding death receptors result in the assembly of death-inducing signaling complexes which initiate the extrinsic pathway by activating caspase-8 [23]. DNA breakage, endoplasmic reticular stress, and growth factor withdrawal are functional signals for activating the release of intrinsic factors, such as cytochrome C and Similar to Mothers Against Decapentaplegic (SMAD), from the inner membrane of mitochondria to trigger the intrinsic pathway [24,25]. The B-cell lymphoma (Bcl)-2 family is composed of pro-apoptotic and anti-apoptotic factors, which manipulate the activity of intrinsic pathway [26]. Several Bcl-2 family genes encode alternatively spliced variants which exhibit pro- or anti-apoptotic activity [27]. The intrinsic pathway-specific apoptosome is assembled to participate in recruiting and processing procaspase-9 [28]. Eventually, processed caspase-9 activates the downstream caspases-3, -6, and -7, which leads to cell apoptosis [29].

Evasion of apoptosis constitutes one mechanism mediating the acquired resistance of cancer cells during treatment with chemotherapeutic agents [30]. Much higher doses of agents are required to achieve efficacy due to the inherent resistance to apoptosis, which induces off-target adverse effects. Therefore, targeting apoptosis toward cancer cells by inducing the extrinsic pathway through TRAIL signaling or eliminating the anti-apoptotic activities of inhibitors of apoptosis, such as Bcl-2, is considered a potential strategy [31,32]. More death receptor-ligand complexes, including TNF receptor (TNFR)-TNF-α, FAS-Fas ligand, TRAIL receptor (TRAILR)1/2 (also referred to DR4/5)-TRAIL were identified as functioning as apoptosis-inducing signal molecules [33,34]. Upon interactions between death receptors and the corresponding ligands, oligomerization and conformation changes of the same receptors expose the cytoplasmic death domain (DD) which is involved in the interaction with other DD-containing proteins [35], subsequently mediating the processing and activation of procaspases [36]. Two alternative splice variants of TRAIL, TRAIL-β, and TRAIL-γ, were identified in neoplastic cells [37]. The lack of exon 3 in TRAIL-β and of exons 2 and 3 in TRAIL-γ, which encode the truncated extracellular binding domain, results in loss of their pro-apoptotic activity [38]. In addition, Bcl-2 family members exhibit both pro- and anti-apoptotic activities on modulating the intrinsic apoptosis pathway [39]. The balance between Bcl-2 family members is meticulously determined by cell survival or apoptosis [40]. Moreover, impaired splicing profiles of Bcl-2 family members result in differential or opposite effects in regulating cell viability [27]. A growing body of studies has demonstrated that predominant expressions of anti-apoptotic members or isoforms participate in the evasion of cancer cells from programmed cell death.

## 3. Overview of Alternative Splicing

**Figure 2:**
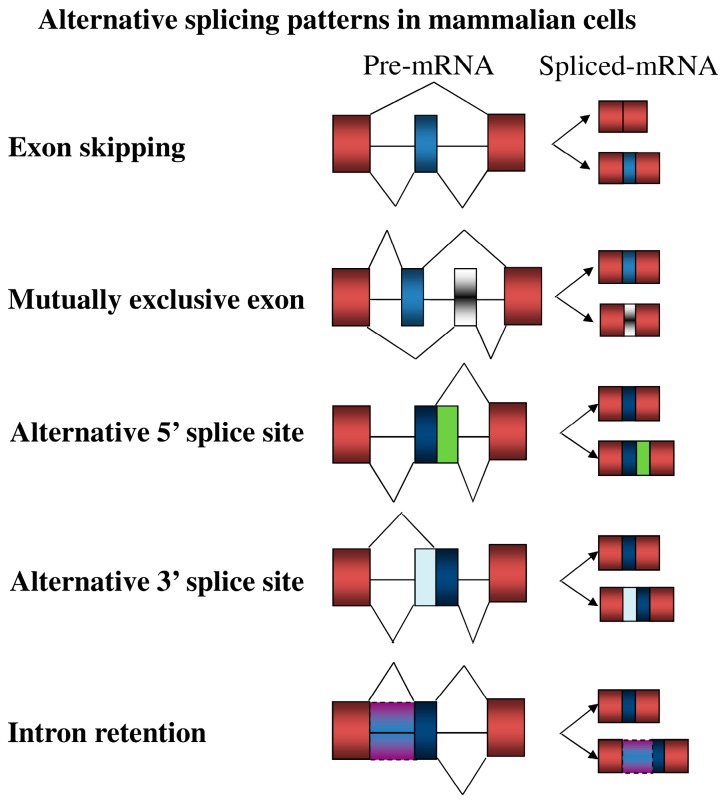
The diagram presents the major alternative splicing modes in mammalian cells.

**Figure 3:**
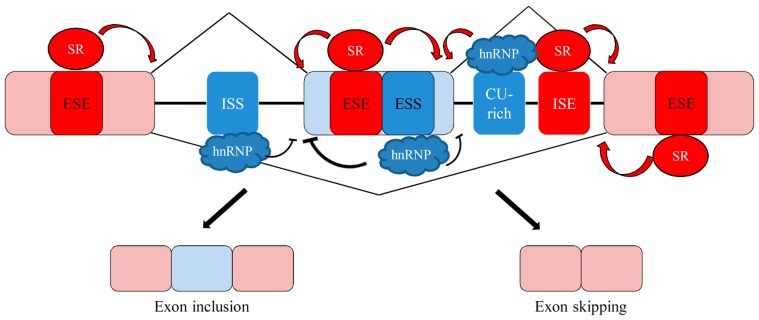
Molecular mechanism involved in alternative splicing of pre-mRNA. The interplay between serine/arginine-rich (SR) proteins and exonic splicing enhancer (ESE) or intronic splicing enhancer (ISE) mostly strengthens the utilization of splice sites. In contrast, the binding of hnRNPs to exonic splicing silencer (ESS) or intronic splicing silencer (ISS) exerts a differential effect on the utilization of splice sites.

Recognition of 5′ and 3′ splice sites is the critical step in the definition of intron in the mammalian genome. The spliceosome is composed of five small nuclear (sn)RNAs and more than 150 associated proteins, which contributes to the splicing of defined introns [41]. However, the utilization of 5′ or 3′ splice sites is widely strengthened or weakened by the interplay between *trans*-splicing factors and the corresponding *cis*-elements within regulated exon and surrounding intron, leading to the alternative splicing regulation in mammalian cells (Figure 2, [42]). The regulatory elements are classified into exonic and intronic splicing enhancers (ESEs and ISEs) or silencers (ESSs and ISSs) according to their impact on the alternatively-spliced exons [43]. Interplay between splicing factors and the binding elements in turn manipulate utilization of 5′ or 3′ splice sites by facilitating or interfering with the assembly of spliceosomes. Heterogeneous nuclear ribonucleoproteins (hnRNPs) and serine/arginine-rich (SR) proteins are two major groups of splicing factors [44]. HnRNPs generally abolish the exon inclusion by binding to the exonic pyrimidine-rich element, whereas the interplay between hnRNPs and intronic binding elements exhibited differential effect on the alternatively-spliced exons [45], (Figure 3). Many studies demonstrated that SR proteins enhance utilization of most alternatively spliced exons by binding to the exonic purine-rich element [45]. However, the interplay between SR proteins and the adjacent exons subsequently mediated the exclusion of internal exons [45], (Figure 3). Collectively, the potential effect of splicing factor on individual splicing event is modulated by sequence or position context that should be further defined. Moreover, splicing profiles are spatial-temporally reprogrammed by the relative expressions of numerous splicing factors in the nucleus [46].

## 4. Impacts of Alternative Splicing Events on Apoptosis

### 4.1. Apoptosis-Related Alternative Splicing Events

#### 4.1.1. Survivin

**Figure 4:**
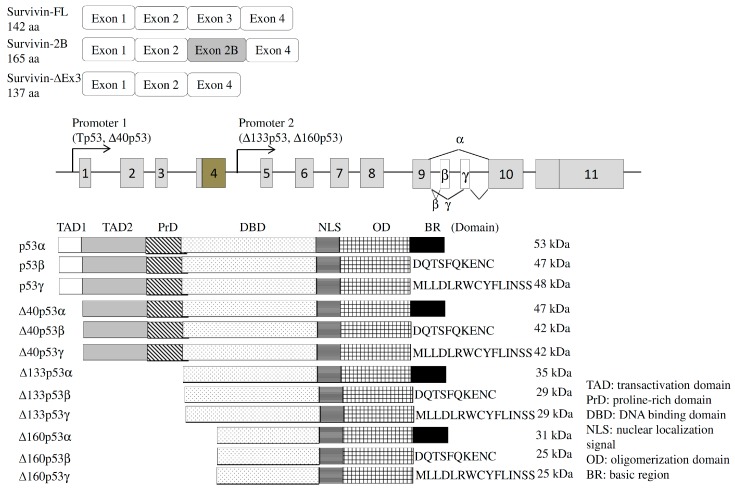
*Survivin* and *p53* genes generated different isoforms from the alternative splicing, usage of alternative promoters, and alternative initiation of translation. Schemes represent the exon composition of *surviving* transcripts (**upper**) and the functional domains of p53 variants (**lower**). DQTSFQKENC and MLLDLRWCYFLINSS: the amino acid sequences of C-terminus of p53β and p53γ isoforms.

Temporal expression of the survivin protein is highly correlated with the transition from the G2/M to the G1 phase [47]. Several reports indicated that ubiquitous expression of the survivin protein in most malignant cells, but not well-differentiated cells, functions as an inhibitor of the apoptosis protein (IAP, [48]). The *survivin* gene produces three alternatively spliced variants, including full length, delta Ex3 (∆Ex3), and 2B transcripts (Figure 4, [49]), which exhibit differential effects on the apoptotic process [50]. Several studies demonstrated the cytoprotective effects of survivin ΔEx3 variants inhibiting the apoptotic process [51]. In contrast, the presence of the survivin 2B isoforms was reported to confer pro-apoptotic properties on cancer cells [51]. Accordingly, relatively high expressions of full length and survivin ∆Ex3 variants are especially correlated with the active progression or poor prognoses of breast, gastric, thyroid, and pituitary cancers [52]. The molecular mechanism involved in programming of the *survivin* gene is largely unclear.

#### 4.1.2. Estrogen Receptor (ER)

The imbalanced stimulation of sexual hormones, including estrogen, frequently leads to breast cancer which is the most common malignancy in females worldwide [53]. ERα and ERβ proteins transmit the action of estrogens into target cells [54,55]. Although generated by individual genes, ERα and ERβ have almost 100% amino acid homology in their DNA-binding domain and about 60% amino acid homology in their protein-interacting domains [56]. Interestingly, in vivo and in vitro experiments demonstrated differential or opposite effects of ERα and ERβ on biological features of breast cancer cells [57]. ERα-regulated gene expressions facilitate the growth and survival of breast cancer cells in response to estrogens [58], whereas the impact of ERβ on breast cancer cells is controversial [59]. hnRNP G and Tra2-β1 proteins were recently demonstrated to modulate the selection of *ERα* exon 7 [60]. Overexpressing Tra2-β1 induced relative levels of *ERα^+ex7^* transcripts, whereas overexpressing hnRNP G exhibited an antagonistic effect on inducing *ERα^−ex7^* levels [60]. Statistical analyses of several cohort studies suggested positive correlations between ERα^+7^ variants and tumor grades in breast cancer [61]. In addition, several orphan receptors that share structural similarities to ERs were characterized as estrogen-related receptors (ERRs). Two alternatively-spliced variants, short-form ERRβ (ERRβsf) and ERRβ2, were recently identified as being involved in cell cycle regulation and survival [62]. Silencing of ERRβ2-suppressed p53 signaling-mediated apoptosis, whereas overexpression of ERRβsf enhanced p21 activity which facilitates cell proliferation [63]. It is widely noted that splice variants often exhibit antagonistic effects. Taken together, these results may bring new insights into the clinical treatment of breast cancer.

#### 4.1.3. Transient Receptor Potential Melastatin (TRPM)

TRPM family members share similar structural signatures, including transmembrane domains and the cytosolic terminus [64]. TRPM members assemble hetero-oligomers to function as a Ca^2+^-permeable cation channel that is related to the progression of malignancies, such as prostate cancer [64]. Among these members, *TRPM3* and *TRPM8* genes reportedly encode variants through an alternative splicing mechanism [65,66]. In addition to full-length transcripts, short *sM8α* and *sM8β* were generated from the *TRPM8* gene through alternative splicing regulation [65]. Spatial expressions of TRPM8 isoforms were noted in lung tissues and prostate cancer [67]. These short transcripts encode the N-terminus region of the TRPM8 protein, and were demonstrated to manipulate the activity and sensitivity of authentic TRPM8 [67]. Overexpression of sTRPM8α, but not sTRPM8β, substantially abolished starvation-induced apoptosis of several prostate cancer cells [66]. Moreover, the presence of sTRPM8α overexpression largely enhanced the activity of metalloproteinase-2, which subsequently induced progression of LNCaP prostate cancer cells [67]. In contrast, the influence of sTRPM8β is largely uncharacterized.

#### 4.1.4. Interleukin (IL)-15

Expression of IL-15 mediates the secretion of inflammatory cytokines which lessens apoptosis of CD8^+^ T cells [68]. Recently, an exon 6-excluded *IL-15* (*IL-15ΔE6*) transcript was identified in lipopolysaccharide-stimulated macrophages and B cells [69]. An in vitro proliferation assay showed that IL-15ΔE6 overexpression interfered with IL-15-induced proliferation of T cells by mediating cell apoptosis [70]. Alternatively-spliced IL-15 variants compete with full-length IL-15 protein for their binding to the IL-15 receptor α [70]. The association between IL15ΔE6 variants and IL-15Rα reduced the maturation and function of macrophages and activated T cells, subsequently reducing the activity of the innate immune system in the central nerve system [70]. Therefore, the presence of IL-15Rα potentially constitutes a regulatory mechanism for manipulating the immune response toward exogenous stimuli.

### 4.2. Alternative Splicing of Apoptotic Factors

#### 4.2.1. Tumor Protein p53 (TP53)

TP53 was documented to be a master factor involved in cell cycle arrest, DNA repair, and apoptosis [71]. Loss of TP53 function was widely discovered in about 50% of human malignancies [71]. In addition to expression levels, alternative splicing regulation constitutes another mechanism for manipulating the effect of the *TP53* gene [72]. The human *TP53* gene was reported to generate 12 isoforms by use of a distinct promoter, translation start site, and alternative exons in normal cells [73]. In brief, three N-terminus variants of human TP53, Δ40TP53, Δ133TP53, and Δ160TP53, were encoded using distinct translation start sites. Three C-terminus domains (α, β, and γ) were differentially selected with four N-terminus regions to generate 12 TP53 variants (Figure 4, [73]). The TP53β variant was documented to enhance the activity of the p21 protein as did TP53β [74]. Moreover, TP53β mediated cell apoptosis through both TP53-dependent and -independent pathways [75]. In contrast, the effect of TP53γ on cell apoptosis was not reported. The association between Δ133TP53α and TP53α was demonstrated to lessen cell apoptosis and cell cycle arrest, which is highly relevant to cancer progression [76].

Reactivation of TP53 is considered a potential gene therapy for TP53-deficient malignancies. A spliceosome-mediated RNA *trans-*splicing (SMaRT) strategy was recently reported to correct the mutant *TP53* gene to the wild-type *TP53* gene through a *trans-*splicing mechanism which involves splicing between two individual transcripts [77]. In brief, the expression plasmid containing a pre-*trans-*spliced exon that encodes the correct TP53 fragment is delivered into *TP53*-defective hepatocellular carcinoma (HCC) cells. Mutant *TP53* transcripts were corrected by replacing the mutant exon with the *trans-*spliced exon, which encoded the functional TP53 protein in *TP53*-defective HCC cells [77]. Introduction of the pre-*trans-*spliced *TP53* exon mediated activation of TP53-responsive genes and subsequently suppressed the progression of HCC cells in vitro.

#### 4.2.2. Fas Signaling

Fas (also referred as Apo-1/CD95) is a well-studied member of the TNF receptor superfamily which mediates extrinsic pathway-induced apoptosis upon interaction with the Fas ligand or agonistic antibodies [78]. Alternative splicing of *Fas* exon 6 generates membrane-bound or soluble isoforms that exhibit opposite activities on cellular apoptosis [79]. Neoplastic cells are frequently noted to reduce Fas expression or induce relative levels of soluble Fas proteins, encoded by *Fas^−exon 6^*, to evade Fas/Fas L-mediated apoptosis [80]. The direct interaction between T-cell intracellular antigen-1 (TIA-1) and the Uridine-rich stretch next to *Fas* exon 6 facilitated the recognition of U1snRNP for the 5′ splice site of *Fas* intron 6 and also enhanced the binding of U2AF to the 3′ splice site of *Fas* intron 5, which led to the definition of *Fas* exon 6 [81]. The binding of polypyrimidine tract binding protein 1 (PTBP1) and the U-rich element (URE) within *Fas* exon 6 exhibited the antagonistic effect on the TIA-1-enhanced inclusion of *Fas* exon 6 [81]. In addition to PTBP1, the direct binding of Hu antigen R (HuR) and the URE within *Fas* exon 6 was demonstrated to reduce the association of U2AF and the 3′ splice site of *Fas* intron 5, subsequently resulting in the skipping of *Fas* exon 6 [82]. The binding of hnRNP C and URE within *Fas* exon 6 cooperatively facilitated the repressive effect of PTBP1 and HuR on interfering with the interaction between TIA-1/TIAR and the 5′ splice site next to *Fas* exon 6 [83]. In addition to the exonic element, recent study indicated that over 90% of single nucleotide mutation (58/63 positions) mediated distinct effect on the usage of *Fas* exon 6 [84], which potentially constituted a novel mechanism regarding the exon definition. Results of genome-wide screening indicated that elimination of more than 200 splicing regulators, including SR proteins, hnRNP family and splicing factor 45, changed the splicing profile of Fas in mammalian cells [85]. In addition, the splicing profile of the *Fas* gene is regulated by natural antisense RNA (*Fas-AS1* or *saf*) which manipulates utilization of *Fas* exon 6 and, therefore, manipulates the proapoptotic activity of Fas signaling [86]. Despite this, the molecular mechanism involved in antisense RNA-regulated splicing is still largely unclear.

**Figure 5:**
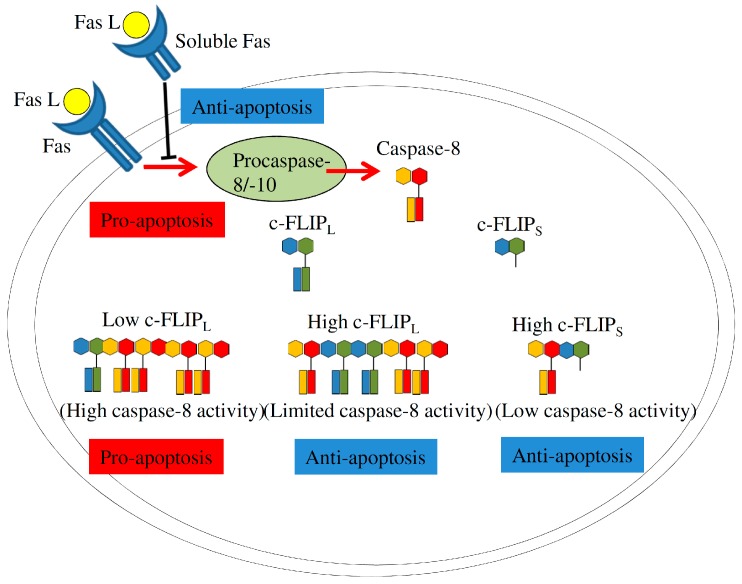
The relative expressions of c-FLIP variants differentially regulate the activity of the caspase-8 pathway, directing the cell fate. Fas L, Fas ligand; c-FLIP, Cellular FLICE inhibitory protein.

Cellular FLICE inhibitory protein (c-FLIP), a caspase-8 homolog, functions as a crucial factor in manipulating apoptotic activity of the Fas/Fas L-mediated pathway [87]. By usage of alternative 5′ splice site within c-FLIP exon 5, the *c-FLIP* gene generates alternative transcripts which encode three variants, including 55 kDa c-FLIP long (c-FLIP_L_), 26 kDa c-FLIP_S_, and 24 kDa c-FLIP_R_, in human cells [88]. The c-FLIP_L_ isoform shares a high homology with procaspase-8 except for a cysteine residue within the catalytic center, whereas short c-FLIP variants are truncated isoforms which lack the dimerization motif of procaspase-8 and only contains the tandem death effector domain (DED) [87]. c-FLIP isoforms exhibit differential effects on restricting activation of procaspase-8. Relatively high expressions of short c-FLIP isoforms substantially interfere with the oligomerization of procaspase-8, resulting in its inactivation and evasion of apoptosis. Overexpressed c-FLIP_L_ isoform assembles heterodimers with procaspase-8, but block its activation [87]. In contrast, at physiological levels, c-FLIP_L_ forms heterodimers with procaspase-8 within the death-inducing signaling complex (DISC), facilitating procaspase-8 activation and subsequent programmed cell death (Figure 5, [87]). Nevertheless, understanding the molecular mechanism involved in the regulation of *c-FLIP* splicing still requires further investigation.

#### 4.2.3. Bcl-2 Family

Bcl-2 family members, including Bcl-2, Bax, Bcl-x, Bcl-g, Bcl-rambo, Bim, Bfl-1, Bid, Mcl-1, and PUMA, are well-characterized factors which exhibit both pro- and anti-apoptotic activities [89]. These proteins form homodimers or heterodimers through Bcl-2 homologous (BH) domains. Therefore, relative levels of Bcl-2 family members are critical in fine-tuning a cell’s fate [90]. In addition, Bcl-2-related genes encode protein isoforms with differential or opposite functions through alternative splicing mechanisms [27].

The *Bcl-x* gene was characterized as generating alternative transcripts by using the alternative 5′ splice site within exon 2, encoding the anti-apoptotic Bcl-x_L_ and pro-apoptotic Bcl-x_S_ isoforms [91]. Relative expressions of these two isoforms manipulate sensitization of mammalian cells under apoptotic conditions [92]. The splicing profiles of *Bcl-x* gene were widely modulated by SR proteins, hnRNP family and RNA binding motif proteins serine/arginine-rich splicing factor 1 (SRSF1), polypyrimidine tract binding protein 1 (PTBP1), RNA binding motif protein 4 (RBM4), RBM5, RBM10, and RBM11, were documented to program the splicing profile of *Bcl-x* [93,94,95]. For example, overexpression of the RBM4 protein mediates relatively high levels of *Bcl-x_S_* transcripts, leading to processing of procaspase-3 and poly(ADP ribose) polymerase (PARP) which function as apoptotic markers [95]. In addition to a splicing regulator, the presence of a splice-switching oligonucleotide (SSO) was also demonstrated to reprogram *Bcl-x* splicing from Bcl-x_L_ to Bcl-x_S_ and subsequently induce apoptosis of human hepatic stellate cells [96]. The antisense oligonucleotide may function as a better therapeutic Bcl-x SSO than other apoptotic inducers that can only focus on splicing mechanisms [96].

Bax was reported to exhibit pro-apoptotic activity [97]. Targeting of dimerized Bax to the mitochondrial membrane resulted in the release of cytochrome C and sequentially induced caspase-9/3-mediated cell apoptosis [98]. It was recently reported that a single guanosine deletion (G8 to G7) within *Bax* exon 3 resulted in the skipping of *Bax* exon 3, which generated the *BaxΔ2* transcript [99]. Interestingly, *BaxΔ2*-positive colorectal cancer (CRC) cells were much more sensitive to adriamycin and 5-FU compared to *BaxΔ2*-negative CRC clones [100]. Moreover, the presence of *the* BaxΔ2 protein further mediated activation of procaspase-8 and downstream apoptotic signaling [100]. However, both CRC clones showed similar sensitivities to treatment with daunorubicin, which shares a structure similar to that of adriamycin. These results suggested that BaxΔ2-positive cells exhibit a preference for specific chemotherapeutic drugs [100].

Mutual utilization of *BIM* exon 3 or 4 constitutes the molecular mechanism involved in the generation of two distinct transcripts [101]. *BIM*^−exon 4^ transcripts encode the BH3 domain-absence variant which exhibited the antagonistic effect toward the activity of anti-apoptotic Bcl-2 proteins [102].The splicing profile of the *BIM* gene is programmed by the interplay between *cis*-acting elements and *trans*-splicing regulators [101]. For example, up-regulated expression of the SRSF1 protein induced a relatively high level of the *BIM*^+exon 3^ isoform in breast cancer cells [103]. Accordingly, overexpression of the SRSF1 protein preferentially lessened the sensitivity of neoplastic cells to chemotherapeutic compound-mediated cell death [103]. In addition, a cytosine-to-thymidine mutation (rs724710) within *BIM* exon 4 was noted to reduce the selection of exon 4 in lymphoblastic leukemia cells, which contributed to drug resistance [104]. Therefore, alternative splicing patterns of the *BIM* gene are considered an emerging mediator of the immortality of cancerous cells. The apoptosis-related splicing events are listed in Table 1.

### 4.3. Splicing Factors Involved in Apoptosis-Related Splicing Events

#### 4.3.1. Serine/Arginine (SR)-Rich Splicing Factors

The serine/arginine-rich splicing factors are widely involved in numerous alternative splicing events [105]. Homozygous knockout embryos of most SR proteins, such as SRSF2, are lethal to embryos, suggesting the important functions that these factors exert in tissue development and organogenesis [106]. Down-regulation of SRSF2 substantially led to cell cycle arrest and destabilization of the genome [107]. Recent reports documented that imbalanced expression of SRSF2 resulted in the reduced growth and imbalanced apoptosis of hematopoietic cells, especially bone marrow cells [108]. Deep RNA-seq results indicated that SRSF2 depletion mediated the aberrant splicing of hematopoiesis-related genes, including *MEIS1*, *UPF38*, *PRKAA1*, *RBM23*, *PDK1*, *PDE4DIP*, *MLL*, and *RNF34*, which are closely related to the homeostasis of myeloid progenitors [108].

SRSF3 reportedly modulated the alternative splicing of several apoptosis-related genes, such as *caspase-2* (*Casp2*), *programmed cell death 4* (*PDCD4*), and *homeodomain-interacting protein kinase-2* (*HIPK2*) in distinct cancer cells [109,110,111]. Caspase-2 was demonstrated to act the early initiator in the intrinsic apoptosis pathway [112]. The utilization of *caspase-2* exon 9 leads to the generation of two caspase-2 isoforms which exert opposite effect on the apoptotic process [112]. The direct interaction between overexpressing SRSF3 and the CU-rich element within *Casp2* exon 8 was reported to promote the skipping of *CASP2* exon 9, which increased the relative expression of anti-apoptotic Casp2_S_ isoform [112]. In distinct colorectal cancer cells, SRSF3 silencing was demonstrated to enhance the selection of alternative 3′ splice site within the *HIPK2* exon 8, generating the 81 nucleotides-deleted HIPK2 Δe8 isoform [110]. HIPK2 Δe8 isoform was resistant to the proteasome-mediated degradation in the absence of E3 ligase binding site which was encoded the deleted 5′ region of *HIPK2* exon 8. The relatively high level of HIPK2 Δe8 isoform profoundly induced the phosphorylation of p53 protein and downstream apoptotic pathway [110]. Moreover, SRSF3 was also reported to modulate the expression of *PDCD4* gene through the alternative splicing and translational mechanisms [111]. PDCD4 reportedly acted the tumor suppressor in repressing the transformation and immortality of cancer cells [113]. SRSF3 silencing was noted to reduce the relative level of *PDCD4* isoform 2, containing the partial *PDCD4* intron 3 in distinct cancer cells. The premature stop codon-harboring *PDCD4* isoform 2 was considered as the potential substrate of nonsense-mediated decay mechanism [46]. Moreover, the preferential binding of SRSF3 with the 5′ UTR of *PDCD4* transcript drove its enrichment to the processing body more than repressed the translational activity of *PDCD4* mRNA. Collectively, elevated SRSF3 expression enhanced the anti-apoptotic signature of cancer cells by reprogramming the splicing profiles of related genes.

SRSF1 (also referred as ASE/SF2) is the well-studied protein involved in most posttranscriptional regulations, including alternative splicing [114]. The manipulated expression of SRSF1 is frequently observed during the organogenesis and carcinogenesis [115]. More than 500 potential candidates, including *insulin receptor* (*INSR*) were identified as the potential candidates of SRSF1 by using the RNA-seq approach [116]. *INSR* gene was demonstrated to generate two alternatively-spliced variants in a spatial-temporal manner [116].The exon 11-excluded *INSR* (*INSR-A*) transcripts were predominantly expressed in embryonic and cancerous cells [116], whereas the exon 11-included *INSR* (*INSR-B*) transcripts were widely noted in the pancreatic β-cells, skeletal muscle, and adipocytes [117]. Overexpression of SRSF1 was demonstrated to constitute a molecular mechanism in enhancing the relative level of exon 11-included *INSR–B*, which subsequently lessened the sensitivity of pancreatic progenitors to stress-induced apoptosis [116]. In addition, the up-regulated SRSF1 expression with a concomitant increase in the relative level of exon-9-included *cancer susceptibility candidate 4* (*CASC4*) transcripts was revealed in breast cancer cells compared to the normal ductal cells [115]. SRSF1 overexpressing cells generated the relatively high level of full-length *CASC4* transcripts which contained the 168-nucleotides long exon 9, encoding the long CASC4 variants [115]. The epithelial MCF-10A ductal cells exhibited progressive and anti-apoptotic activity in the presence of exogenous CASC4-FL protein, whereas the overexpressing CASC4-Δ9 variant exerted limited effect on these signatures [115].

#### 4.3.2. Heterogeneous Nuclear Ribonucleoprotein (hnRNP) Family

The hnRNP family is composed of about 20 members that play significant roles in transcriptional, post-transcriptional, and translational regulation [118]. Several hnRNP proteins are considered to be proto-oncogenes according to recent studies [119]. Among these members, hnRNP A1, hnRNP K, and PTBP1 (hnRNP I) are involved in apoptosis-related splicing events. The expression of hnRNP A1, an hnRNP family member, is abundant and ubiquitously generated [120]. A bioinformatics analysis and RNA-protein binding assay indicated a direct interaction between hnRNP A1 and *Fas* exon 5, which subsequently facilitated inclusion of *Fas* exon 6 [121]. Due to the proapoptotic activity of *Fas^+exon 6^*, using hnRNP A1 and other splicing regulators may be considered a potential strategy to reduce the immortality of cancer cells. HnRNP K was reported to strengthen utilization of the 5′ splice site by interacting with an exonic enhancer and subsequently interfered with the generation of *Bcl-x_S_* transcripts, which led to evasion of apoptosis by cancerous cells [122]. In addition, the association of hnRNP K with the Sam68 protein abrogated its effect in inducing relative levels of *Bcl-x_S_* transcripts [123]. Nevertheless, the hnRNP K protein diminishes apoptotic activity through multi-layer mechanisms [124]. The PTBP1 was demonstrated to facilitate the Warburg effect in colorectal cancer cells by reprogramming splicing profiles of the *PKM* gene [125]. Recent studies indicated the emerging role of PTBP1-modulated regulation in diminishing cell apoptosis with treatment with an antitubulin agent [126]. The presence of PTBP1 reduced the stability of *Mcl-1* transcripts by binding to its 3′ UTR [126]. Moreover, the ablation of PTBP1 induced the apoptotic evasion of antitubulin-treated cells in a Mcl-1-dependent manner [126]. However, the influence of PTBP1 on other post-transcriptional mechanisms, such as alternative splicing regulation, in terms of Mcl-1 isoform expressions is worthy of further investigation. In addition, overexpressing PTBP1 functioned as a splicing silencer of *Bim* exon 3 by directly targeting the responsive element within *Bim* intron 2 [101].

#### 4.3.3. RNA-Binding Motif Proteins (RBMPs)

RBMPs constitute another family that participates in diverse gene regulation. Individual RBMPs contain multiple RNA recognition motifs (RRMs) which are the most common class of RNA-binding domains. RNA-binding motifs are multi-functional and have been implicated in nucleotide- and protein-protein interactions of RBMPs. The binding surface of RRMs is composed of 80–90 residues which are folded in four-strand anti-parallel β-sheets [127]. In addition, the serine/arginine rich elements were widely noted as well in various RBMPs. Among the RBMPs, RBM4, 5, 10, and 11 were demonstrated to modulate apoptosis-related splicing events in various malignancies [95,128,129]. RBM4 was shown to program splicing cascades which are closely relevant to the development of the mesodermal lineage, including skeletal muscles and brown adipocytes [130]. Recent reports indicated the tumor-suppressive effect of RBM4 through regulating splicing profiles of apoptosis-related genes [95]. Relatively low levels of RBM4 were noted in cancerous tissues compared to adjacent normal tissues which were dissected from non-small cell lung cancer (NSCLC) and breast cancer (BC) patients [95]. Overexpression of RBM4 increased the relative ratio of *Bcl-x_S_* transcripts and subsequently induced apoptosis of several lung cancer cell lines [95]. Moreover, the association of overexpression of RBM4 with *Mcl-1* exon 2 and intron 2 shifted *Mcl-1_L_* to *Mcl-1_S_* transcripts, which, in part, deprived breast cancer cells of apoptotic resistance against chemotherapeutic treatment [131]. RBM5 and RBM10 are highly similar homologues which share about 50% amino acid identity [132]. Previous studies showed the regulatory effect of RBM5 on modulating splicing profiles of *c-FLIP*, *Fas*, and *caspase-2* [129,133]. Reduced expressions of RBM5 and RBM10 were noted in cancerous tissues of NSCLS, prostate cancer, and BC patients compared to adjacent normal counterparts [132]. Overexpression of RBM5 or RBM10 both consistently resulted in relatively high levels of *Fas^−exon 6^* transcripts which encoded soluble and antiapoptotic isoforms in different cells [134]. Recent studies showed that RBM5 and RBM10 exhibited similar effects on the same apoptosis-related splicing events, such as *c-FLIP*, *caspase-2*, *caspase-3*, *caspase-9*, and *Bcl-x* genes [129,133]. However, the differential influence of each RBM5/10-modulated splicing event on cell apoptosis was individually characterized. The *endocytic adaptor protein* (*NUMB*) gene was identified as a novel candidate of RBM5, RBM6, and RBM10 [132]. NUMB has been reported to participate in the activation of p53 protein by regulating the Notch pathway [135]. Intriguingly, depletion of RBM5 and RBM10 showed opposite effects on inclusion of *NUMB* exon 9. Subsequently, RBM5 and RBM10 exhibited differential influences on the radiosensitivity and proliferation of lung adenocarcinoma cells through NUMB-mediated Notch signaling [132]. Collectively, cell apoptosis and proliferation are meticulously controlled processes which are regulated through multilayer mechanisms. The apoptosis-related splicing regulators are summarized in Table 2.

## 5. Conclusions and Perspectives

Alternative splicing was demonstrated to be an important molecular mechanism that is widely involved in the homeostasis of mammalian cells. Dysregulated splicing events were widely demonstrated to be molecular hallmarks of developmental and malignant diseases. In this review, we attempted to summarize recent studies regarding the influence of alternatively spliced transcripts on cell apoptosis, which is highly relevant to organogenesis and carcinogenesis. The impacts of splicing regulators on apoptosis-related splicing events were discussed as well. Along with the development of high-throughput approaches, including deep RNA sequencing and proteomics analyses, new insights will be brought to the identification of disease-associated splicing networks on a genome-wide scale. A thorough realization of the mechanisms underlying development- and cancer-related splicing networks will function as a convincing source of therapeutic strategies for treating inherited and malignant diseases.

## Figures and Tables

**Table 1:** Exon/intron usage and biological relevance of apoptosis-related alternative splicing (AS) events.

Gene	AS Region	AS Type	Splicing Regulator	Biological Signatures	Reference
*Survivin*	Exon 3 Exon 2B	Exon skipping Exon skipping	uncharacterized	anti-apoptosis (Survivin ∆Ex3 and 3β) pro-apoptosis (Survivin 2β and 2α)	[49,50,51,52]
*ERa* *ERb*	Exon 7 Exon 10/11	Exon skipping Exon skipping	hnRNP G, Tra2b unclear	anti-apoptosis (ERα^+7^) pro-apoptosis (ERRb2) anti-apoptosis (ERRβsf)	[60,61,62,63]
*Transient receptor potential melastatin 8*	Exon 5a	Exon skipping	uncharacterized	anti-apoptosis (sTRPM8α)	[65,66,67]
*Interleukin-15*	Exon 6	Exon skipping	uncharacterized	pro-apoptosis (IL-15ΔE6)	[69,70]
*p53*	Exon β/γ	Exon skipping	uncharacterized	pro-apoptosis (p53β variants)	[72,73,74,75,76]
*Fas*	Exon 6	Exon skipping	TIA-1/TIAR, PTBP1, HuR, hnRNP C	anti-apoptosis (Fas^−exon 6^) pro-apoptosis (Fas)	[79,80,81,82,83,84,85]
*c-FLIP*	Exon 5	Alternative 5′ SS	RBM5/10	pro-apoptosis (c-FLIP_L_) anti-apoptosis (c-FLIP_S_)	[87,88]
*Bcl-x*	Exon 2	Alternative 5′ SS	SRSF1, PTBP1, RBM4, RBM5, RBM10, and RBM11	anti-apoptosis (Bcl-x_L_) pro-apoptosis (Bcl-x_S_)	[91,92,93,94,95,96]
*Bax*	Exon 3	Exon skipping	uncharacterized	pro-apoptosis (Bax and BaxΔ2)	[99,100]
*BIM*	Exon 3/4	Mutual selection	SRSF1	pro-apoptosis (BIM^+exon 3^) anti-apoptosis (BIM^+exon 4^)	[101,102,103,104]

**Table 2:** Distinct splicing factors modulate a set of apoptosis-related alternative splicing (AS) events.

Splicing Regulator	Specific Candidate	Impact on AS	Biological Signatures	Reference
SRSF1	*INSR*	Exon 11 inclusion (*INSR-B*)	anti-apoptosis	[115,116,117]
	*CASC4*	Exon 9 inclusion	anti-apoptosis	
SRSF3	*Casp2*	Exon 9 skipping	anti-apoptosis	[109,110,111,112,113]
	*HIPK2*	Alternative 3′ SS selection (Exon 8)	pro-apoptosis	
	*PDCD4*	Intron retention (Intron 3)	anti-apoptosis	
HnRNP A1	*Fas*	Exon 6 inclusion	anti-apoptosis	[121]
HnRNP K	*Bcl-x*	Authentic 5' SS selection (*Bcl-x_L_*)	anti-apoptosis	[122,123,124]
HnRNP I	*Mcl-1*	Exon 2 inclusion (*Mcl-1_L_*)	anti-apoptosis	[126]
	*Bim*	Exon 4 inclusion	anti-apoptosis	[101]
RBM4	*Bcl-x*	Alternative 5′ SS selection (*Bcl-x_S_*)	pro-apoptosis	[95]
	*Mcl-1*	Exon 2 skipping (*Mcl-1_S_*)	pro-apoptosis	[131]
RBM5/10	*c-FLIP*	Alternative 5′ SS selection	anti-apoptosis (c-FLIP_S_)	[129]
	*Fas*	Exon 6 skipping	anti-apoptosis	[134]
	*Casp2*	Exon 9 inclusion	pro-apoptosis	[133]
	*NUMB*	Exon 9 inclusion/exclusion	uncharacterized	[132]
